# Does early application of needle-knife sphincterotomy (NKS) in patients with difficult biliary cannulation increase the risk of postERCP pancreatitis? A single centre study

**DOI:** 10.12669/pjms.39.3.6777

**Published:** 2023

**Authors:** Rao Saad Ali Khan, Laima Alam, Farrukh Saeed, Farrukh Sher, Rao Zaid Ali Khan

**Affiliations:** 1 Rao Saad Ali Khan, FCPS Med, FCPS Gastroenterology, FRCP Consultant Gastroenterology and Transplant Hepatologist, Pak Emirates Military Hospital, Rawalpindi, Pakistan; 2 Laima Alam, FCPS Gastroenterology, MRCP (UK), CHPE Consultant Gastroenterology, Bahria International Hospital, Rawalpindi, Pakistan; 3 Farrukh Saeed, FCPS Med, FCPS Gastroenterology. Head of Department of Gastroenterology, Pak Emirates Military Hospital, Rawalpindi, Pakistan; 4 Farrukh Sher, FCPS Med. Fellow Gastroenterology, Pak Emirates Military Hospital, Rawalpindi, Pakistan; 5 Rao Zaid Ali Khan, Research Assistant, Pak Emirates Military Hospital, Rawalpindi, Pakistan

**Keywords:** Endoscopic Retrograde Cholangiopancreaticography, Guide-wire cannulation, Needle-knife sphicterotomy, Post-ERCP pancreatitis, Endoscopic sphincterotomy

## Abstract

**Objective:**

To determine that early needle-knife sphincterotomy does not increase post-ERCP pancreatitis in patients with difficult biliary cannulation as compared to standard cannulation.

**Method:**

This prospective single-centre cohort study was carried out at Pak Emirates Military Hospital from January 2021 to June 2021. Patients requiring ERCP were enrolled in the study (according to inclusion and exclusion criteria) and were subsequently allotted different groups according to the technique used for deep biliary cannulation. Qualitative data was analysed using frequencies and chi square statistics whereas, quantitative data was analysed using mean±SD and one way ANOVA test.

**Result:**

The cohort included 114 patients with 52.6% male patients and predominance of relatively younger age group (31-45 years). The most common indication for ERCP was choledocholithiasis (36%) with an overall technical success rate of 96%. Deep cannulation was achieved either through standard cannulation (56%), double guidewire and/or pancreatic stent assisted (10.5%), use of early Needle-Knife Sphincterotomy (19%), NKS as a last resort (3.5%) or Transpancreatic Stenting and/or combined sphincterotomy (6%). Pancreatitis as a complication occurred in 4(3.5%) patients, bleeding in 2(1.8%), on-table desaturation in 2(1.8%) and perforation in 1(0.9%) patient. The occurrence of pancreatitis was only related significantly to inadvertent PD cannulation through univariate and logistic regression analysis whereas, multiple cannulations (>5), gender, age, classification of papilla and the use of early NKS had no impact on pancreatitis or the occurrence of other complications.

**Conclusion:**

NKS is an effective and safe modality for deep biliary cannulation and achieving technical success where cannulation is deemed difficult and does not increase the risk of PEP if done by experienced endoscopists in high volume centres.

## INTRODUCTION

Although endoscopic retrograde cholangiopancreatography (ERCP) has become an important standard procedure for the management of pancreatic biliary diseases, the failure to achieve deep biliary cannulation still ranges from 5% to 18% with 2% to 10% risk of post-ERCP pancreatitis (PEP) as a substantial adverse event.^1^ There is a continuous conscious effort to develop effective techniques to improve biliary cannulation success rate along with minimizing intra and post procedure complications. Some of the extensively studied techniques are needle knife sphincterotomy (NKS), double-guidewire technique, trans-pancreatic sphincterotomy, combined sphincterotomies and EUS guided procedure.^2^

Needle-knife sphicterotomy (NKS) has long been considered a risk factor of PEP, perforation and bleeding in literature and has been historically tried only as a last resort.^1^-^3^ However multiple recent studies have reported its efficacy and safety if implemented early on when performed by experienced endoscopists at high volume centres.^4^ The confounders like repetitive attempts at cannulation, inadvertent pancreatic duct (PD) instrumentation and ampullary edema secondary to excessive manipulation were found to be responsible for the high rates of PEP in these studies.^5^ Thus, NKS can be considered an independent marker of a lengthy and complex ERCP.

A thorough review of the literature revealed that only two studies have been conducted from Pakistan regarding the safety profile of NKS in terms of complications.^6^,^7^ and no study was found to discuss secondary outcomes like failure rates, use of other specialized biliary cannulation techniques, procedure time and relation of failure with modifiable and non-modifiable factors. This study was designed to report the relation of PEP with multiple patient and procedural factors including the use of early NKS (as one of the most important study outcome) by minimizing operator bias. A detailed account of other biliary cannulation techniques as well as the relation of failure rate was extensively studied in this single-centre research project.

## METHODS

This study was designed as a prospective single-centre cohort study carried out at Pak Emirates Military Hospital, Rawalpindi from January 2021 to June 2021 after obtaining ethical committee review (A/28/EC/407/2022, dated Nov 2021) and patients’ consent.

All patients, age 15 years and above with indication for ERCP and consenting to the study were enrolled. Exclusion criteria included age less than 15 years, non-consenting individuals, active shock and hemodynamic instability, coagulopathy, severe cardio-pulmonary disease and pregnancy.

### Procedure:

The study was performed in a dedicated tertiary care advance GI procedure suite by two high volume Consultant Endoscopists with 90% success rate of biliary cannulation and over 400 ERCPs per year. A strict cannulation protocol of a total of less than five cannulation attempts with no more than two inadvertent PD cannulations was followed. In some of the cases, the anatomy of the papilla compelled the endoscopists to resort to early NKS. If deep cannulation was not achieved through either of the methods; double guidewire and/or pancreatic stent assisted procedure or TPS was tried according to the indication of the procedure, the local anatomy, patient dynamics and operator’s discretion.

Both the operators used the same technique for deep cannulation and the demographics, extensive cannulation data, laboratory and outcome measures were noted for every patient.

All patients were observed for six hours post-ERCP and those with significant abdominal pain were retained for 24 hours.^8^ All the patients were given prophylactic indomethacin rectally prior to the procedure.

### Definitions:

*Cannulation attempt* was defined as a continuous contact between the papilla and the sphincterotome for at least five seconds.^8^ Resorting to NKS after a total of less than five attempts at cannulation with standard method was defined as *early NKS*. In some of the cases, NKS was tried earlier at the endoscopist’s discretion considering the local anatomy. In other cases, *NKS as the last resort* was tried after five or more unsuccessful attempts with standard method or using guidewire assisted procedures. *Difficult biliary cannulation* was defined as more than five attempts at papillary cannulation, more than five minutes spent to cannulate after papilla was clearly visualized or more than one inadvertent PD cannulation.^9^ *Technical success* was defined as free and deep instrumentation of the biliary tree.^8^ *Total time of the procedure* was defined as the time from initial intubation to the procedure termination^1^. *Pancreatitis* was defined as abdominal pain and more than three times rise in serum amylase levels 24 hours post-procedure. *Cholangitis* was defined as fever of >38^°^C with abdominal pain for more than 24 hours.^10^ Perforation was defined as the presence of free air or contrast leakage seen radiogarphically.^1^

### Outcomes:

Primary outcomes for our study included technical success and the occurrence of complications. Secondary outcomes included total procedure time, time to deep cannulation and goal attainment, inadvertent PD cannulation, PD stenting and number of attempts at cannulation.

### Statistical analysis:

Sample size was calculated through OpenEpi sample size calculator with 80% power by using the postulation that PEP incidence is 5 to 20% with ERCP.^11^ Qualitative data was represented as frequencies and analysed using Chi square test. Quantitative data was analysed using mean±SD and one way ANOVA test. The relation between pancreatitis as well as that of failed procedures with different factors was seen through univariate and logistic regression analysis. All data was analysed using SPSS V.21 with p value <0.05 considered significant.

## RESULTS

A total of 114 patients were enrolled according to the inclusion criteria with 60 (52.6%) males and majority of the patients (42%) belonging to the age group 31 to 45 years. Fifty-three (46.5%) of the patients had no prior co-morbidities and 69 (60.5%) belonged to ASA-I class (Table-I). The most common indication of ERCP was choledocholithiasis (36%), followed by benign CBD strictures (20%), stent replacement (13%) and hilar malignant strictures (8.6%).

**Table-I:** Demographics of the cohort (n=114).

Variables	Primary success (n=76)		Secondary success (n=33)			Failed (n=5)

	Standard cannulation (n=64)	Double guidewire and/or pancreatic stent assisted (n=12)	Early NKS (n=22)	NKS as last resort (n=4)	TPS and/or combined sphicterotomy (n=7)	
Age (years)						
15-30	4(3.5)	5(4.4)	2(1.8)	0	3(2.6)	0
31-45	26(23)	5(4.4)	11(9.6)	4(3.5)	1(0.9)	1(0.9)
46-60	15(13)	2(1.8)	6(5.3)	0	1(0.9)	1(0.9)
61-75	16(14)	0	1(0.9)	0	0	1(0.9)
>75	3(2.6)	0	2(1.8)	0	2(1.8)	2(1.8)
Male gender	39(34)	2(1.8)	13(11)	2(1.8)	4(3.5)	2(1.8)
***Comorbidities***						
None	27(24)	10(9)	10(9)	4(3.5)	4(3.5)	2(1.8)
Diabetes Mellitus	5(4.4)	0	6(5.3)	0	0	2(1.8)
Hypertension	20(17.5)	2(1.8)	7(6)	0	0	3(2.6)
COPD	3(2.6)	0	1(0.9)	0	0	1(0.9)
CKD	2(1.8)	0	0	0	0	0
CLD	1(0.9)	0	0	0	0	0
Obesity	2(1.8)	0	1(0.9)	0	0	0
Post liver transplant	1(0.9)	0	1(0.9)	0	0	0
Ulcerative colitis	1(0.9)	0	0	0	0	0
***ASA***						
I	35(31)	11(9.6)	13(11.4)	3(2.6)	5(4.4)	2(1.8)
II	20(17.5)	1(0.9)	6(5.3)	1(0.9)	1(0.9)	1(0.9)
III	9(8)	0	3(2.6)	0	1(0.9)	2(1.8)

The laboratory parameters are shown in Table-II. Technical success was achieved in 109 (96%) of the patients with deep cannulation performed either through standard cannulation (56%), double guidewire and/or pancreatic stent assisted (10.5%), use of early NKS (19%), NKS as a last resort (3.5%) or TPS and/or combined sphincterotomy (6%). Multiple cannulation was only seen in 9(8%) cases of which, two procedures eventually failed and had to be referred for percutaneous trans-hepatic biliary dilatation (PTBD) (Table-III).

**Table-II:** Laboratory parameters and papillary anatomy of the cohort analysed (mean±SD or n%).

Parameters	Primary success (n=76)		Secondary success (n=33)			Failed (n=5)	P value

	Standard cannulation	Double guidewire and/or pancreatic stent assisted	Early NKS	NKS as a last resort	TPS and/or combined sphicterotomy		
TLC	9.6±2.6	7.3±1.4	8.9±2.6	8.3±2.7	9.6±2.3	11±1.5	0.044
ALT	109±72	72±28	79±7	55±22	79±32	83±28	0.007
AST	118±78	78±29	85±33.5	60±24	109±33.5	89±27	0.116
Bilirubin	56±39	32±11	62±37	26±11	44±37	119±37	≤0.001
Alkaline Phosphatase	336±188	283±74	306±170	189±134	275±238	505±113	0.106
GGT	161±86	134±62	149±62	86±79	121±119	230±8	0.073
Amylase						
-At 6 hours	123±99	259±48	179±226	219±221	187±137	124±39	0.093
-At 24 hours	142±229	344±400	229±371	347±502	174±118	98±39	0.193
Papillary anatomy							
-Regular	42(37)	11(9.6)	11(9.6)	3(2.6)	6(5.3)	1(0.9)	
-Protruding	18(16)	1(0.9)	7(6)	0	0	3(2.6)	0.017*
-Peri/intra-diverticular	0	0	4(3.5)	1(0.9)	1(0.9)	1(0.9)	
-Surgically altered anatomy	4(3.5)	0	0	0	0	0	

* Chi square.

**Table-III:** Outcome of ERCP in the study population (n(%) or mean±SD)

Outcome measures	Primary success		Secondary success			P value

	Standard cannulation	Double guidewire and/or pancreatic stent assisted	Early NKS	NKS as last resort	TPS and/or combined sphicterotomy	
Total time of procedure (sec)	1326±65	2000±189	1560±112	2715±376	2169±222	≤0.001
Time for localization of papilla (sec)	35.5±5	26.6±1.4	39.5±5	32±9.5	32±5	0.883
Time for deep cannulation (sec)	251±406	113±357	212±445	271±406	77±334	≤0.001
Time for acquiring goal (sec)	727±744	194±628	404±784	627±544	131±532	≤0.001
PD cannulation	4(3.5)	11(9.6)	5(4.4)	3(2.6)	6(5.3)	≤0.001*
PD stenting	0	7(6)	0	0	4(3.5)	≤0.001*
Technical success	64(56)	12(10.5)	22(19)	4(3.5)	7(6)	≤0.001*
***Number of attempts at cannulation***						
5	64(56)	9(8)	22(19)	0	7(6)	≤0.001*
>5	0	3(2.6)	0	4(3.5)	0
***Complications***						
None	59(52)	11(9.6)	21(18.4)	2(1.8)	6(5.3)	0.001*
Bleeding	1(0.9)	0	1(0.9)	0	0	
Perforation	0	0	0	1(0.9)	0	
Pancreatitis	1(0.9)	1(0.9)	0	0	1(0.9)	
Desaturation	1(0.9)	0	0	0	0	

* Chi square.

Total time of the procedure varied among the groups significantly, owing to the fact that highly specialized cannulation techniques were used only after standard cannulation was tried and deemed unsuccessful. In this study, pancreatitis as a complication occurred in 4(3.5%) patients (all of whom were treated conservatively except one who required EUS guided cyst gastrostomy), bleeding in 2(1.8%) patients and on-table desaturation in two patients (requiring neck extension and high flow oxygen). Perforation occurred in one patient when NKS was tried as a last resort and the patient had an uneventful recovery through conservative management. The patients that underwent early NKS showed almost negligible complications with only a single patient presenting minor bleed that required adrenaline injection. The mortality rate was zero for our study with meticulous follow up of the cases with complications (Table-III).

The occurrence of pancreatitis was only related significantly to inadvertent PD cannulation through univariate and logistic regression analysis whereas, multiple cannulations (>5), gender, younger age (<60 years), classification of papilla and the use of early NKS had no impact on pancreatitis or the occurrence of other complications (Table-IV). An analysis of the failed procedures revealed correlation with old age (>60 years) through univariate and logistic analysis but showed significance only towards malignant biliary strictures as an indication through multinomial logistic regression (Table-V). Also, cannulation attempts of four or more were significantly related to failure rate through multinomial regression analysis.

**Table-IV:** Analysis for the occurrence of PEP as a complication in relation to multiple factors.

Factors	Univariate analysis	Binary logistic regression
Age (≤60 vs >60 years)	0.261	0.254
Gender	0.347	0.236
Presence of comorbidities	0.423	0.430
PD cannulation	0.001	0.001
Prophylactic PD stent placement	0.634	0.294
Multiple attempts at cannulation (≤5 vs >5)	0.20	0.232
Early NKS	0.324	0.317
Papilla (Normal vs Abnormal)	0.437	0.99

**Table-V:** Analysis of procedure failure in relation to multiple factors.

Factors	Univariate analysis	Binary logistic regression
Age (≤60 vs >60 years)	0.036	0.035
Gender	0.513	0.509
Classification of papilla (normal vs abnormal)	0.427	0.423
Presence of comorbidities	0.487	0.483
Multiple attempts at cannulation	0.006	0.022^£^
Indication	0.006	*
ASA	0.195	0.130
Occurrence of complications	0.66	0.663

A cannulation attempts of four and more times were significantly related to failure through multinomial regression analysis.

* showed no significance through binary logistic regression but only significant for hilar and distal malignant strictures through multinomial logistic regression analysis.

## DISCUSSION

Post ERCP pancreatitis (PEP) has been extensively studied over the recent years due to an increase in demand of pancreatico-biliary disease management. Sphincter of Oddi dysfunction (SOD), female gender, relatively young age, non-dilated CBD, inadvertent PD cannulation, multiple cannulation attempts in lieu of difficult biliary cannulation, contrast injection and a previous history of PEP have all been reported to be significant factors contributing towards PEP.^12^

Fistulotomy and sphincterotomy have long been considered contributors to PEP and were only used as the last resort. However, this has been rigorously challenged and many researchers now believe that papillary and PD edema secondary to excessive manipulation is the reason for pancreatitis and NKS itself is a surrogate marker for difficult cannulation.^1^ Interestingly, it has been seen that an early application of NKS can actually reduce the overall risk of complications, especially PEP from 6.1% to 3.9%.^13^

In our study NKS was not found to be responsible for PEP through univariate and logistic regression analysis. Although the risk of PEP was low for our study (3.5%) the only factor that did contribute to the occurrence of PEP was inadvertent PD cannulation. The rate of PEP for our study was comparable to many large-centre randomized studies^14^ and the fact that pre-cut is not associated with increased risk of PEP was also supported by multiple similar studies.^1^,^12^-^17^ However, a local study reported pre-cut and NKS as a significant contributor towards PEP with 64% of the total PEP cases being directly related to pre-cut sphincterotomy.^6^

This raises the question whether pre-cut PEP is operator dependent and if yes, whom should be allowed to do biliary sphincterotomies? A study by Li et al concluded that on average, 13 sessions of NKS were required to achieve a success rate of 85% and 50 sessions were required to reduce the rate of complications below 5%.^18^

The only other complication that was seen for our cohort undergoing early NKS was minor bleeding in a single case that needed intra-procedure adrenaline injection. Also the time required for deep cannulation was less when early NKS was applied as compared to standard cannulation. It is worthwhile to mention that younger age (<60 years) and female gender were not statistically significant for PEP in our study which might be due to non-matched nature of the sample. Another local cross-sectional study with 200 participants reported CBD perforation and hypoxia to be significantly related to NKS^7^, although no such relation was seen for our study.

This study also showed that the anatomy of papilla has no correlation with post-sphincterotomy complications nor with the success rate. Similar results have been reported by Park CS et al by studying 154 NKS cases with 33 cases presenting with peri-ampullary diverticulae.^19^ Multiple attempts at cannulation (>5) was also not found to be statistically significant for our study population, probably due to the fact that only nine cases underwent multiple cannulation attempts and the cases that seemed to be difficult from initial scrutiny were switched to the early NKS or TPS group.

### Limitations:

The study has its limitations including single-centre non-randomized design, non-consideration of SOD as a risk factor for PEP and a non-matched sample. The strengths of the study include extensive data collection, removal of operator bias and follow up of cases for complications that might appear late.

## CONCLUSION

This study concludes that NKS is an effective and safe modality for deep biliary cannulation and achieving technical success where cannulation is deemed difficult and does not increase the risk of PEP if done by experienced endoscopists in high volume centres.

### Authors’ contribution:

**RSAK** contributed to the idea, data collection, procedure, patient care and critical review.

**LA** contributed to the study design, pro forma, statistical analysis and drafting of the manuscript. She is also responsible for the integrity and accuracy of the study.

**FS** contributed to patient care and critical review.

**FS** contributed to patient care and data collection.

**RZAK** contributed to literature review and statistical analysis.
